# Asymptomatic infection with clade 2.3.4.4b highly pathogenic avian influenza A(H5N1) in carnivore pets, Italy, April 2023

**DOI:** 10.2807/1560-7917.ES.2023.28.35.2300441

**Published:** 2023-08-31

**Authors:** Ana Moreno, Francesco Bonfante, Alessio Bortolami, Irene Cassaniti, Anna Caruana, Vincenzo Cottini, Danilo Cereda, Marco Farioli, Alice Fusaro, Antonio Lavazza, Pierdavide Lecchini, Davide Lelli, Andrea Maroni Ponti, Claudia Nassuato, Ambra Pastori, Francesca Rovida, Luigi Ruocco, Marco Sordilli, Fausto Baldanti, Calogero Terregino

**Affiliations:** 1 Istituto Zooprofilattico Sperimentale della Lombardia e dell’Emilia Romagna (IZSLER), Brescia, Italy; 2 Istituto Zooprofilattico Sperimentale delle Venezie (IZSVe), Legnaro (Padua), Italy; 3 Department of Clinical, Surgical, Diagnostic and Paediatric Sciences, University of Pavia, Pavia, Italy; 4 SC Microbiology and Virology, IRCCS Policlinico San Matteo, Pavia, Italy; 5 General Directorate of Welfare, Regione Lombardia, Milan, Italy; 6 Sanità Animale e Farmaci Veterinari, Ministero della Salute, Rome, Italy; 7 Dipartimento Veterinario e Sicurezza degli Alimenti di origine animale-ATS Brescia Direzione Generale, Brescia, Italy; 8 Dipartimento di Igiene e Prevenzione Sanitaria-ATS Brescia, Brescia, Italy; * These authors contributed equally to this work and share last authorship.

**Keywords:** highly pathogenic avian influenza H5N1 virus, PB2 mutation, silent infection, pet carnivores, phylogenetic analysis, serology

## Abstract

In April 2023, an outbreak of clade 2.3.4.4b highly pathogenic avian influenza A(H5N1) viruses carrying the T271A mammalian adaptive mutation in the PB2 protein was detected in a backyard poultry farm in Italy. Five domestic dogs and one cat living on the premises had seroconverted in the absence of clinical signs. Virological and serological monitoring of individuals exposed to the virus proved the absence of human transmission, however, asymptomatic influenza A(H5N1) infections in mammalian pets may have important public health implications.

Since autumn 2021, highly pathogenic avian influenza A (HPAI) H5N1 clade 2.3.4.4b viruses have been detected in several continents [1] with a several spill-over events in mammals, which have raised concern about the ability of these viruses to infect and adapt to humans. The polymerase activity of avian influenza viruses (AIVs) is a known determinant of viral fitness. However, it is still unclear why the polymerase activity of viruses of avian origin is limited in mammalian cells. The adaptation of avian viruses to mammals, through natural selection processes leading to adaptive mutations in polymerase proteins, is an essential factor in increasing its replicative capacity in mammals [2,3]. Here we report a case of influenza A(H5N1) infection in a domestic cat and five dogs living on a rural backyard poultry farm where an HPAI H5N1 outbreak was notified; the infection in poultry was caused by an HPAI H5N1 virus strain belonging to the BB genotype that was characterised by the presence of a PB2 mutation related to mammalian adaptation.

## Case description and sampling

On 17 April 2023, increased mortality was recorded in a backyard poultry farm in the province of Brescia (northern Italy). As required by current Italian legislation, a suspicion of avian influenza was raised following the notification to the authorities; the farm was quarantined, and samples were taken to be tested for HPAI. At the time of quarantine, 22 poultry were still alive on the farm: two adult chicken hens, 10 chicks, eight ducks and two geese. Mortality was recorded only in the hens, with 16 dead animals among the original 18 hens on the farm. Deceased birds presented only cyanosis of the crest and wattles. 

The farm is in the vicinity of several water sources, such as ditches and a fishing pond (< 300 m away), at a distance of ca 10 km south of Lake Garda. On the farm lived five domestic medium-sized crossbreed dogs and one cat that were allowed to roam freely inside and outside the farm. These animals did not present any clinical signs at the time mortality was observed for hens, nor in the following weeks. 

**Figure 1:**
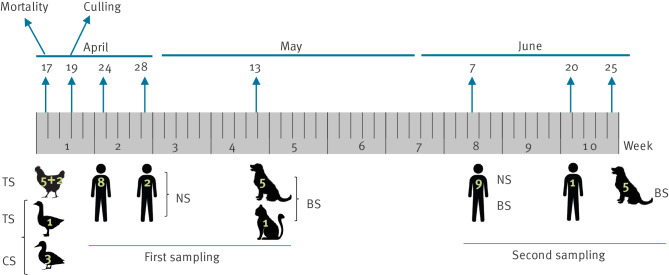
Timeline and description of samples collected, highly pathogenic avian influenza A(H5N1) infections, Italy, April 2023

BS: blood samples; CS: cloacal swabs; NS: nasal swabs; TS: tracheal swabs.

The sampling protocols and timing are summarised in Figure 1. Upon detection of an HPAI H5 virus, additional sampling was planned for exposed humans and mammals on the farm according to the timelines shown in Figure 1. 

Numbers in yellow represent the number of collected samples. In hens, five samples were collected from live and two from dead animals. Samples were analysed in pools of up to five swabs for detection avian influenza virus genome. Samples taken from humans and other mammals were analysed individually.

In the first sampling, nasal swabs were collected from 10 humans (mean age: 59.5 years; range: 3–93) who were either residents of the farm or had occupational exposure, while nasal swabs and blood from the same individuals were collected for a second sampling 6–9 weeks later. Among those sampled were the owner, their five family members and four professionally exposed persons, in particular three veterinarians and one worker involved in bird culling and carcass disposal.

In dogs, double sampling was also performed but only of blood, given the time elapsed since the outbreak. Unfortunately, only the first sampling could be performed for the cat because it was absent on the day of the second sampling, although it always remained clinically healthy and was present on the farm on the following days.

## Virological investigations

Tracheal and cloacal swabs from birds and samples from humans were analysed for detection of the AIV genome using a real-time RT-PCR targeting the M gene [4]. Positive samples were then analysed with two real-time RT-PCRs for the detection of the H5 (protocol code PDPVIR143) [5] and H7 (PDPVIR144) [4] genome, as well as for neuraminidase subtype and H5 pathotype by two real-time RT-PCR specific for N1 (PDPVIR1004) [6] and for the H5-specific multi-basic HP cleavage site (PDVVIR1005) [7]. Tracheal swabs from the hens resulted positive for AIV and were characterised as H5N1 HPAI viruses, whereas the cloacal swabs from the ducks and the goose and all samples from humans resulted negative in all tests.

**Figure 2:**
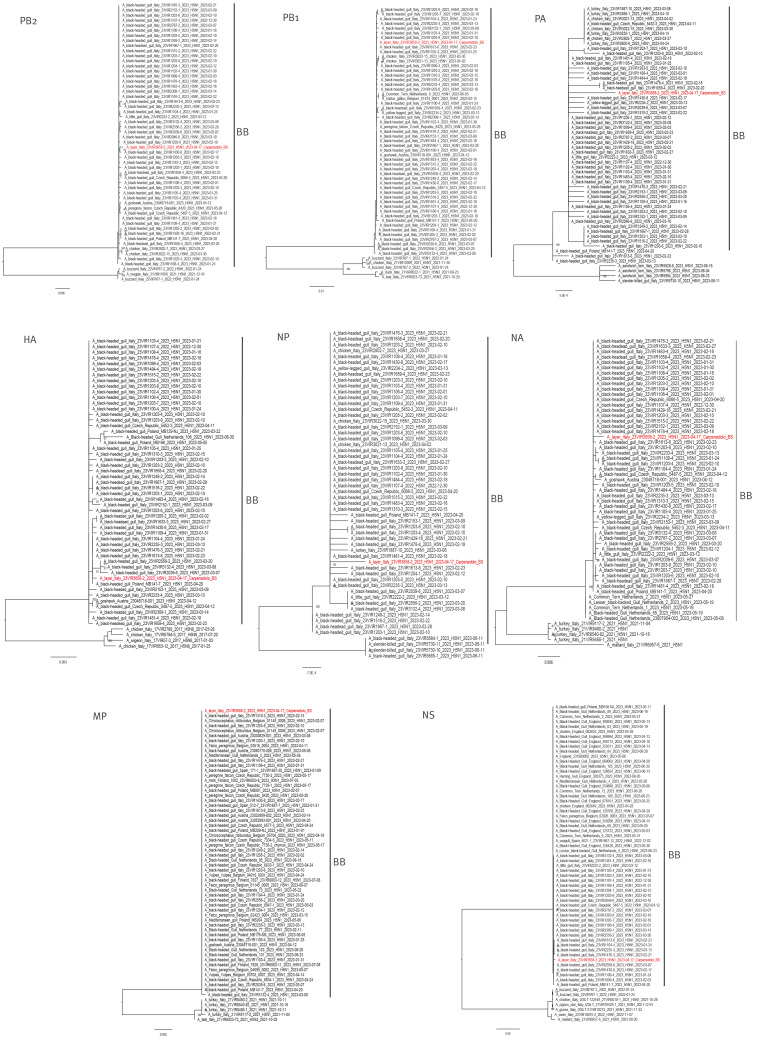
Maximum likelihood phylogenetic trees of all gene segments, highly pathogenic avian influenza A(H5N1) virus sequences from one hen, Italy, April 2023

Complete genome sequencing of one positive sample was done with next generation sequencing according to a protocol modified from [8]. The sequences were aligned against the most related sequences identified from a BLAST search in GISAID (Table 1). The topology of the phylogenetic trees of the eight gene segments indicates that the virus belongs to clade 2.3.4.4b, genotype BB (H5N1-A/Herring_gull/France/22P015977/2022-like) [9] and clusters together with the H5N1 viruses collected in black-headed gulls (*Chroicocephalus ridibundus*) in Italy during the first months of 2023 (Table 1, Figure 2). The molecular analysis identified an unusual mutation in the PB2 protein, T271A, which is a marker of virus adaptation to mammalian species [2,10].

**Table 1:** BLAST search of highly pathogenic avian influenza A(H5N1) virus sequences performed in the GISAID EpiFlu database, April 2023

Gene	Segment	% identity	GISAID accession number	Virus strain
PB2	1	99	EPI2559964	A/black-headed_gull/Italy/23VIR1109–4/2023 (A(H5N1)
PB1	2	99	EPI2559949	A/black-headed_gull/Italy/23VIR1104–4/2023 (A(H5N1)
PA	3	99	EPI2559963	A/black-headed_gull/Italy/23VIR1109–4/2023 (A(H5N1)
A HA	4	99	EPI2559951	A/black-headed_gull/Italy/23VIR1104–4/2023 (A(H5N1)
NP	5	99	EPI2559960	A/black-headed_gull/Italy/23VIR1109–4/2023 (A(H5N1)
NA	6	99	EPI2559966	A/black-headed_gull/Italy/23VIR1109–4/2023 (A(H5N1)
MP	7	100	EPI2559962	A/black-headed_gull/Italy/23VIR1109–4/2023 (A(H5N1)
NS	8	99	EPI2559961	A/black-headed_gull/Italy/23VIR1109–4/2023 (A(H5N1)

Source: http://www.gisaid.org.

Generated in IQTREE v1.6.6 [23]. Sequences of the virus sequenced in this study are shown in red.

## Serological testing

Serum samples from the dogs and the cat were analysed for the detection of antibodies against nucleoprotein type A (NPA), haemagglutinins H5, H7 and neuraminidases N1, N2, N3 and N7 using different methods.

Sera were first tested for anti-NPA antibodies using two monoclonal antibody (MAb)-competitive ELISAs to detect anti-NPA antibodies, namely an in-house assay (NPA-EL) and the ID Screen Influenza A Antibody Competition Multi-species (ID-VET-NPA; Innovative Diagnostics). Two further in-house MAb-based competitive ELISAs were used to detect H5 (H5-EL) [11] and H7 [12] antibodies. The presence and specificity of H5 antibodies were confirmed by the hemagglutination inhibition (HI) test performed according to the World Organisation for Animal Health Terrestrial manual [7] and by microneutralisation (MN) assay performed in a biosafety level 3 laboratory [13] using two different viruses based on their genetic and antigenic characteristics: HPAI-H5N1 A/Gull/Italy/23VIR1310–12/2023 (Gull/23) and LPAI-H5N3 A/Mallard Duck/Netherlands/38/2008 (Mal/08) (kindly provided by Ron Fouchier, Erasmus Medical Center, Rotterdam, the Netherlands), belonging to the Goose/GD lineage clade 2.3.4.4b and the Eurasian lineage, respectively. Finally, the presence of antibodies against N1, N2, N3 and N7 was investigated using previously described MAb-based competitive ELISAs [14]. 

Serological examinations detected antibodies against NPA, H5 and N1 in all sera collected from the dogs and the cat at the two sampling times. Results are summarised in Table 2. Moreover, to further evaluate the specificity of the HI and MN assays, archive sera from dogs (n = 10) and cats (n = 10) collected during routine diagnostic activities were screened against the H5N1 Gull/23 antigen; these yielded negative results. Sera from the first sampling resulted positive by HI assay against the H5N1 Gull/23 virus (> 1:10) in all animals aside from one dog. The HI titres ranged between 1:10 and 1:40. The MN assay against the same strain recorded higher titres (1:80–1:320), and all animals were positive. In contrast, when Mal/08 virus was used for the HI and MN assays, titres were either lower or negative in most animals. In particular, all sera tested by the HI assay using Mal/08 gave negative results, while only the cat and one of the dogs scored neutralising titres of 1:20 and 1:40 in the MN assay using Mal/08, respectively. Interestingly, these two subjects had previously been identified as the animals recording the highest HI and MN titres against Gull/23. 

**Table 2:** Serological results from dog and cat samples, outbreak of highly pathogenic avian influenza A(H5N1), Italy, April 2023 (n = 6)

	First serum collection (13 May, 26 days after outbreak)											Second serum collection (23 June, 66 days after outbreak)	
Method	C-ELISA		Microneutralisation		Haemagglutination inhibition		C-ELISA					C-ELISA	
Cut-off	% inhibition		Serum dilution				% inhibition					% inhibition	
	> 55%^a^	≥ 75%^b^	≥ 1:10^c^				≥ 75%^b^					≥ 75%^b^	
Target antibodies	NPA		H5					N1	N2	N3	N7	NPA	N1
ID	ID-VET-NPA	NPA-EL	HPAI H5N1 Gull/23^d^	LPAI H5N3 Mal/08^d^	HPAI H5N1 Gull/23^d^	LPAI H5N3 Mal/08^d^	H5-EL	N1-EL	N2-EL	N3-EL	N7-EL	NPA-EL	N1-EL
Cat 1	P	P	1:160	1:20	1:40	N	P	P	N	N	N	NP	NP
Dog 1	P	P	1:80	N	1:40	N	P	P	N	N	N	P	P
Dog 2	P	P	1:40	N	N	N	P	P	N	N	N	P	P
Dog 3	P	P	1:40	N	1:10	N	N	P	N	N	N	P	P
Dog 4	P	P	1:80	N	1:10	N	P	P	N	N	N	P	P
Dog 5	P	P	1:320	1:40	1:20	N	P	P	N	N	N	P	P

C-ELISA: competitive ELISA; EL: various C-ELISAs; HPAI: highly pathogenic avian influenza; ID: identification number; ID-VET-NPA: Innovative Diagnostics Screen Influenza A Antibody Competition Multi-species C-ELISA; LPAI: low pathogenic avian influenza; N: negative; NP: not performed; NPA: nucleoprotein A; OD: optical density; P: positive.

^a ^Cut-off: OD value reduction expressed as % inhibition respective to the OD negative sample (OD Sample/OD Negative %).

^b^ Cut-off: OD value reduction expressed as % inhibition respective to the reference value of 100% control wells.

^c^ Cut-off value for establishing the positivity of HI and MN assays.

^d^ Virus used as antigen or test strain.

Assays used: C-ELISA for detection of antibodies against NPA in multi-species: ID-VET-NPA; NPA-EL (Innovative Diagnostics, kit IZS-BS); C-ELISA for detection of antibodies against H5 in multi-species: H5-EL (kit IZSBS) [11]. C-ELISAs for the detection of antibodies against N1, N2, N3 and N7 respectively of avian IAV in multi-species (kits IZSBS): N1-EL; N2-EL; N3-EL; N7-EL [14].

Sera from the 10 exposed humans were tested for H5 antibodies using the same H5-EL competitive ELISA used for the pets and an MN assay that was methodologically identical to the assay deployed to test the pet sera, but differed in the selection of the test virus HPAI-H5N1 A/duck/Italy/326224/2/22VIR909/2022 (Duck/22), a strain that belonged to the 2.3.4.4b clade and antigenically matched circulating BB genotype viruses (data not shown). All 10 humans were asymptomatic at the time of the two draws. Both serum samples gave negative results with both methods (Table 3).

**Table 3:** Anamnestic data, influenza A real-time RT-PCR and serological results from human samples, outbreak of highly pathogenic avian influenza A(H5N1), Italy, April 2023 (n = 10)

Subject	Age (years)	Date of first nasal swab	PanFluA RT-PCR	Date of second nasal swab and blood sample	PanFluA RT-PCR	C-ELISA; H5-EL	MN HPAI H5N1 Duck/22^a^
			Cut-off > 40 Cq		Cut-off > 40 Cq	% inhibition, cut-off ≥ 75%	Serum dilution, cut-off ≥ 1:10
#1	3	24 Apr	N	7 Jun	N	N	N
#2	32	24 Apr	N	7 Jun	N	N	N
#3	93	24 Apr	N	7 Jun	N	N	N
#4	50	24 Apr	N	7 Jun	N	N	N
#5	69	24 Apr	N	7 Jun	N	N	N
#6	38	24 Apr	N	7 Jun	N	N	N
#7	62	24 Apr	N	7 Jun	N	N	N
#8	61	28 Apr	N	7 Jun	N	N	N
#9	58	28 Apr	N	7 Jun	N	N	N
#10	67	24 Apr	N	20 Jun	N	N	N

Cq: quantification cycle; C-ELISA: competitive ELISA; EL: various C-ELISAs; HPAI: highly pathogenic avian influenza; MN: microneutralisation; N: negative.

^a^ Virus used as test strain in the MN assay.

## Discussion

Here we report the serological evidence of HPAI H5N1 virus infection in five dogs and one cat on a rural farm in Italy. The virus identified in hens on the same farm, potentially responsible for the transmission of the virus to pet carnivores, belonged to the BB genotype, which emerged in Europe in May 2022 from reassortment events with the gull-adapted H13 subtype viruses, from which PA, NP and NS genes were acquired. In Italy, genotype BB was first identified in January 2023 and has spread widely in black-headed gulls in northern Italy, causing mass mortality events around Lake Garda in the same geographical area when the affected farm is placed [9]. This genotype was also responsible for seven outbreaks on commercial farms in the Veneto and Emilia Romagna regions [9]. Hence, its detection in this backyard farm in the Lombardy region was not unexpected, given its wide distribution among wild birds in the area surrounding the farm.

It was surprising to observe that the virus characterised in this study, detected in hens, differed from all other HPAI A(H5N1) clade 2.3.4.4b viruses circulating in poultry and in birds by a mutation in the PB2 protein, T271A, which is a marker of virus adaptation to mammalian species; it has previously been shown to be associated with increased polymerase activity in mammalian cells [2,10] and is present in the 2009 pandemic A(H1N1) virus [2]. It should be noted that this mutation has never been observed in H5Nx viruses of clade 2.3.4.4b collected from birds in Europe since 2020. In contrast, it has been detected in ca 7% of clade 2.3.4.4b viruses identified in mammals in Europe, including the virus responsible for the outbreak on a mink farm in Spain [15]. This molecular finding suggests that virus spread from mammals to birds cannot be excluded.

The recent cases of influenza A(H5N1) virus infections in domestic cats in France [16] and Poland [17] and in farmed fur animals across Finland [18] were initially detected because of their overt clinical manifestations characterised by severe respiratory distress and neurological signs; similar presentations are commonly associated with H5N1 cases in wild mammals [9]. In contrast, the affected pets in this report were completely asymptomatic, raising concerns over the possibility of subclinical infections with zoonotic viruses in animals in close contact with humans. The presence of antibody titres against HPAI H5 between 1:40 and 1:320 in the MN test suggests true infection rather than just exposure to the antigen. These results are in line with the recently published study by Chestakova et al. on a high number of HPAI H5 virus infections and antibodies in wild carnivores in the Netherlands during 2020–2022 [19]. In that study, antibody titres against HPAI H5 from 1:20 to 1:160 by HI test were detected and were considered indicative of natural infection even in the absence of clinical symptoms. In addition, experimental challenge of Beagles with an H5N8 virus of clade 2.3.4.4b by nasal route presented a pathogenetic and clinical picture compatible with that observed in our case series, as the dogs showed few or no signs of infection with low nasal viral shedding, seroconversion with low HI titres, and transmission of infection to a sentinel contact [20].

The infection and transmission routes, as well as the pathogenicity of influenza A(H5N1) viruses in farmed and pet carnivores are still poorly understood. To improve our surveillance strategy and preparedness, further serological surveys and experimental research are needed to fully understand the ecology of H5N1 viruses in these animals.

Following the evolution of the epidemiological situation for HPAI and in light of the increasing signs of the circulating viruses’ adaptation to mammals, the Italian Ministry of Health has prepared, in the event of an outbreak of HPAI, specific surveillance plans for exposed people (Ministerial circular 0056437–08/12/2021-DGPRE-DGPRE-P) [21] and domestic carnivores (Device 0009342–04/04/2023 - DGSAF-MDS-P) [22] through syndromic, virological and serological surveillance.

## Conclusions

This study highlights the importance of genetic surveillance to promptly detect viruses with increased zoonotic potential. Complete genomic sequencing of viruses is essential to identify the presence of gene mutations correlated with an adaptation of avian viruses to mammals.
